# Establishing an Immune System Conferring DNA and RNA Virus Resistance in Plants Using CRISPR/Cas12a Multiplex Gene Editing

**DOI:** 10.1002/pld3.70070

**Published:** 2025-04-07

**Authors:** Lili Luo, Liqing Miao, Xuhui Ma, Jinjin Hu, Suzhen Li, Wenzhu Yang, Shuai Ma, Rumei Chen, Xiaoqing Liu

**Affiliations:** ^1^ Biotechnology Research Institute Chinese Academy of Agricultural Sciences Beijing China; ^2^ Key Laboratory of Crop Biology of Anhui Province Anhui Agricultural University Hefei China

**Keywords:** BSCTV, CRISPR‐Cas12a, multiplex gene editing, TMV, virus resistance

## Abstract

Two types of CRISPR/Cas systems (Cas9 and Cas13) have been used to combat eukaryotic viruses successfully. In this study, we established resistance to the DNA virus BSCTV and RNA virus TMV in *Nicotiana benthamiana* using the CRISPR‐Cas12a multiplex gene editing system. We employed two effector proteins LbCas12a and FnCas12a coupled with six guide RNAs targeting virus genome and a novel mRNA–gRNA nucleic acid complex to transport gRNA efficiently. Compared with the BSCTV accumulation in the wild‐type *N. benthamiana* , it was reduced by more than 90% by most transgenic events derived at 7 days post‐inoculation. Additionally, the shoot‐tip leaves were normal in the transgenic plants, whereas they appeared severely curled and stunted in wild‐type *N. benthamiana* at 15 days post‐infection. Target sites evaluation revealed that the editing system can directly destroy the structure of BSCTV viral genomes via large fragment deletions. We quantified TMV virus accumulation in the transgenic *N. benthamiana* lines by monitoring dynamic changes in GFP fluorescence and quantitative analysis by qPCR showed that the CRISPR‐Cas12a system can introduce TMV virus resistance to *N. benthamiana* by preventing its systemic spread. Our study provides an innovative strategy—an mRNA–gRNA nucleic acid complex—which has proven to be highly effective in the gene‐editing system and offers an efficient antiviral approach for generating virus‐resistant plants.

## 1 Introduction

The CRISPR‐Cas system, an adaptive immune mechanism evolved in prokaryotes to combat mobile genetic elements, has been repurposed into a programmable genome‐editing tool. While its initial characterization in *Streptococcus thermophilus* revealed CRISPR‐Cas9's role in bacterial immunity (Barrangou et al. 2007), the seminal work by Doudna, Charpentier, and colleagues established its in vitro DNA cleavage activity (Jinek et al. 2012). Subsequent adaptations by Zhang and Church groups enabled efficient genome editing in eukaryotic systems (Cong et al. 2013), laying the foundation for its transformative applications across genetic engineering. There are numerous CRISPR‐Cas systems found in nature, which can be broadly categorized into two classes. In Class 1 system, complexes consisting of multiple Cas‐effector proteins are necessary to cleave the foreign nucleic acid, while Class 2 requires only a single Cas effector protein to accomplish it (Makarova et al. 2020). Members of each group are further divided into six types according to different modes of immune action. The former includes Type I, Type III, and Type IV, while the latter includes Type II, Type V, and Type VI (Zhang 2019). The CRISPR‐Cas9 system of Type II system has been used more and studied more deeply. CRISPR‐Cas9 is the third generation of genome site‐directed editing technology after zinc finger endonuclease (ZFN) and transcription activator effector nuclease (TALEN) (Gaj et al. 2013). This technology has wide applications and extremely high commercial value. Gao's lab has successfully used CRISPR‐Cas to modify the DNA of plants, applying CRISPR‐Cas to the field of plant biology research (Shan et al. 2013). Furthermore, genome editing technologies enable the modification of endogenous plant genes or exogenous nucleic acid to achieve diverse improvements in agronomic traits, including morphological traits (plant height, leaf angle), seed composition (starch types, protein content and composition), nutritional enhancement (enrichment of aromatic compounds and trace elements), and disease resistance (viral, bacterial, and fungal pathogens).

Viral diseases, the second most common diseases in plants after fungal diseases, reduce crop quality and yield. Because viruses depend on the host cell's machinery to multiply and spread, the prevention and control of viral diseases is more difficult than for other diseases. The use of various transformation methods to produce virus‐resistant plants has been well characterized in the literature. Engineering virus resistance in plants began by demonstrating a delay in disease development in transgenic *Nicotiana benthamiana* plants expressing the coat protein (CP) gene from *Tobacco masaic virus* (TMV) (Abel Patricia et al. 1986). With the development of genome editing biotechnology, CRISPR‐Cas systems have been used to produce antiviral plants. CRISPR‐Cas9–derived antiviral strategies have targeted *Beet severe curly top virus* (BSCTV) by inhibiting virus accumulation (Ji et al. 2015), *Bean yellow dwarf virus* (BeYDV) by slowing its replication speed, thereby reducing the viral genome copy number (Baltes et al. 2015), and *Cucumber mosaic virus* (CMV) and TMV by binding viral RNA with FnCas9–sgRNA complex to block viral genome replication and protein translation (Zhang et al. 2018). CRISPR‐Cas13a antiviral strategies have targeted TMV in *N. benthamiana* , *Southern rice black‐streaked dwarf virus* (SRBSDV) in rice and *sweet potato chlorotic stunt virus* (SPCSV) (Aman et al. 2018; Yu et al. 2022; Zhang et al. 2019). A new strategy that combines CRISPR‐Cas13a with a *Sweet potato leaf curl virus* (SPLCV) replicon‐based expression system might significantly improve resistance to RNA viruses. The SPLCV replicons could replicate and increase the copy number of carried DNA and which leads to high expression levels of Cas13a and sgRNA; this can enhance the efficiency of CRISPR‐Cas–mediated gene editing (Yu et al. 2020).

In this study, we established resistance to the DNA virus BSCTV and RNA virus TMV in *N. benthamiana* using the CRISPR‐Cas12a (Cpf1) multiplex gene editing system. Cas12a is a dual‐nuclease capable of cleaving both DNA (Zetsche et al. 2015) and RNA (Fonfara et al. 2016). Utilizing sgRNA‐Cas to specifically target sequences in the viral genome proved to be an effective method for generating RNA virus–resistant plants (Zhang et al. 2018). Transgenic *N. benthamiana* plants containing the CRISPR‐Cas12a (Cpf1) multiplex gene editing system exhibited significantly reduced virus infection symptoms and lower accumulation of both BSCTV and TMV. Evaluation of target sites in BSCTV‐infected *N. benthamiana* plants revealed a variety of mutations in the BSCTV genome. This study pioneers a dual‐functional CRISPR‐Cas12a system conferring simultaneous resistance to DNA (BSCTV) and RNA viruses (TMV) in plants, surpassing single‐target Cas9/Cas13 systems. We engineered a self‐processing mRNA–gRNA complex with enhanced nuclear export efficiency and a six‐gRNA multiplex strategy inducing structural viral genome collapse via large deletions. Spatiotemporal optimization enabled coordinated Cas12a activity across nuclei (pre‐crRNA processing) and cytoplasm (RNA virus targeting). We believe that the strategy outlined here could be beneficial for developing resistance to multiple agricultural plant viruses.

## 2 Results and Discussion

### 2.1 Designing Gene Editing Constructs for Anti‐BSCTV in *N. benthamiana*

**FIGURE 1:**
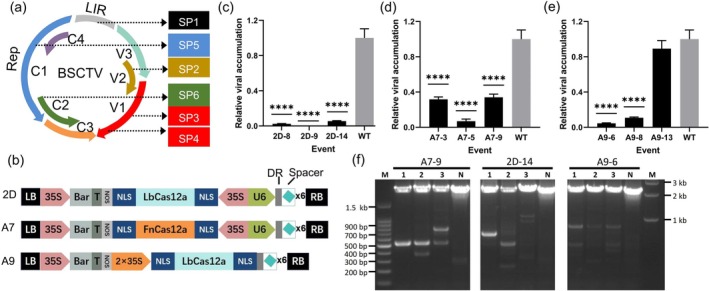
Studies of the strategy of plant anti DNA virus based on CRISPR/Cas12a. (a) Schematic diagram of the BSCTV genome and gRNA target sites. SP1–SP6, sgRNA target sites; LIR, transcriptional regulation region. (b) Schematic diagram of the gene editing vectors for BSCTV. DR, direct repeat; × 6, six pre‐gRNAs made of six DRs with six different spacers respectively. (c–e) qPCR quantification of BSCTV accumulation. WT, BSCTV‐infected wild type *Nicotiana benthamiana* . (f) Mutation detection in the target sites by PCR. N, negative material isolated from transgenic material. The presence of more than two bands in each lane of the transgenic sample indicates the diversity of mutations in the BSCTV genome. Error bars represent the standard deviation; **p < 0.01; ***p < 0.001; ****p < 0.0001. Data are representative of four biological replicates.

BSCTV is a single‐stranded (ss)DNA virus, and its double‐stranded (ds)DNA intermediate needs to be formed in the nucleus where it then produces new ssDNA through rolling circle replication. The most efficient way to inhibit BSCTV accumulation is to target dsDNA intermediates to prevent virus replication (Ji et al. 2015). Hence, the sequence selection for SP1, SP3, and SP5 of BSCTV was based on literature‐reported target sites (A3, B7, and C3) with demonstrated efficacy in disease resistance (Ji et al. 2015), and we selected adjacent sequences to these regions. SP2 targets the ORF V2 on the viral strand, which is involved in regulating viral genome levels and movement. SP4 targets the ORF V1 on the viral strand, encoding the coat protein critical for viral packaging and movement. SP6 targets the ORF C1 on the complementary strand, encoding the Rep protein (Replication initiator protein), which initiates viral genome replication. Therefore, we designed three constructs (2D, A7, and A9) containing two effector proteins from *Lachnospiraceae bacterium* ND2006 (LbCas12a) and *Francisella novicida* U112 (FnCas12a) coupled with six guide RNAs (gRNAs) targeting the BSCTV genome: SP1 targets the replication initiation site, SP2 targets the open reading frame (ORF) of V2, SP3 and SP4 target the CP, and SP5 and SP6 target Rep (Figure 1a). Because Cas12a could recognize direct repeat (DR) sequence of CRISPR RNA for self‐splicing to release mature gRNA, we employed the strategy that produce six gRNAs from one multi‐gRNA cassette in all constructs (Figure 1b). The difference between construct 2D and A7 is that the species origin of Cas12a protein is different. Both of them use the constitutive promoter 35 s to drive the expression of Cas12a protein gene with nuclear localization signal (NLS) peptide, and use the U6 promoter to drive the transcription of multi‐gRNA cassette (Figure 1b). In order to make full use of the ability of Cas12a protein to process its own CRISPR RNA, we fused the multi‐gRNA cassette to the 3′ end of the LbCas12a coding region in construct A9 (Figure 1b). Therefore, some of the fused transcripts could leave the nucleus for Cas12a translation. Then, Cas12a entered the nucleus to process the precursor CRISPR RNA under the guidance of the NLS.

### 2.2 Generation of Transgenic *N. benthamiana* and Characterization of Its Antiviral Properties

**FIGURE 2:**
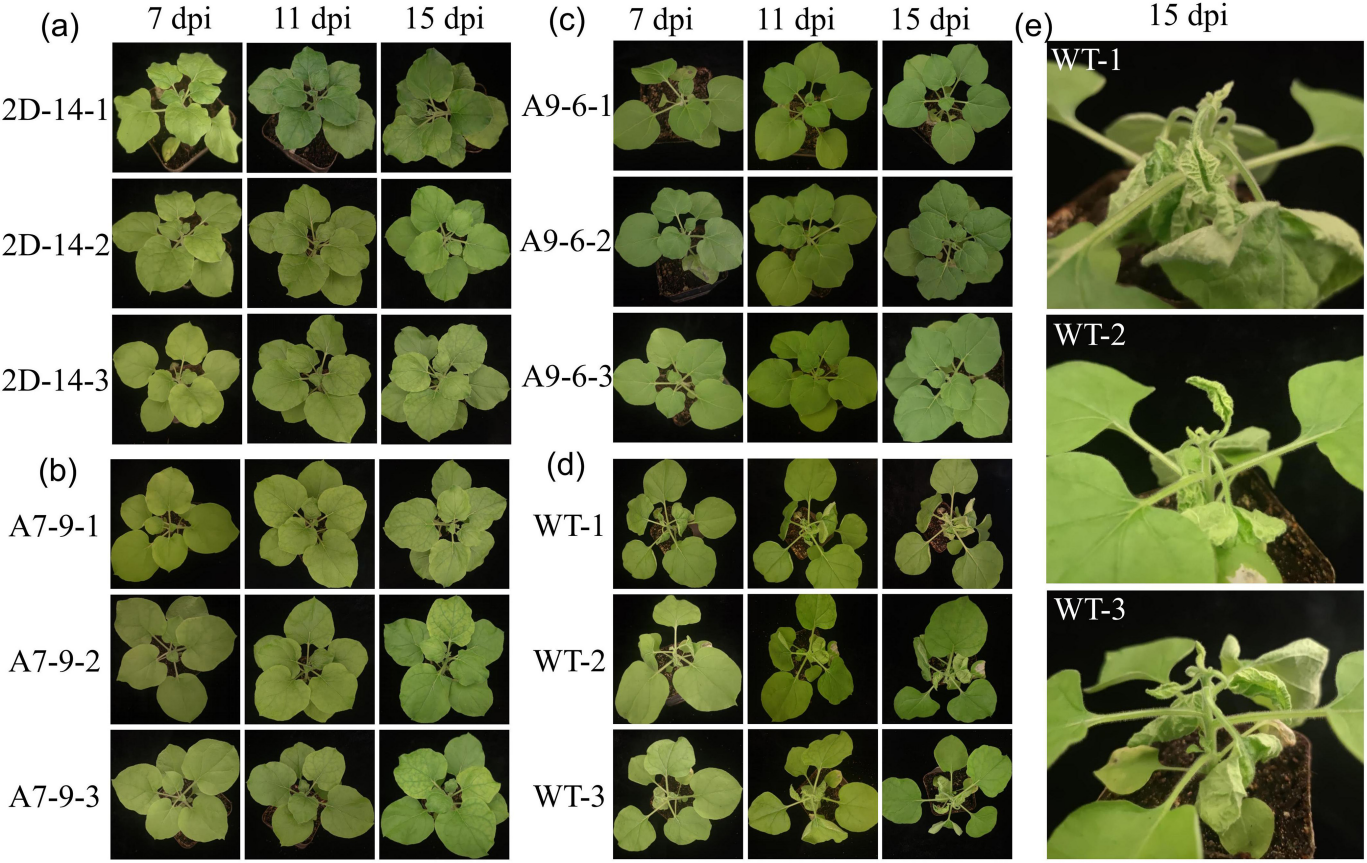
Transgenic *Nicotiana benthamiana* plants established resistance to BSCTV. The symptoms of T2 transgenic *N. benthamiana* plants after BSCTV inoculation were monitored. Three T2 individuals from each of the three gene editing constructs derived transgenic *N. benthamiana* and the WT were selected to observe the symptoms during 15 dpi. No typical BSCTV symptoms appeared on the infected transgenic *N. benthamiana* plants (a–c) during the 15 dpi, while leaf curling was observed on the infected WT plants early at 7 dpi (d), and more severely at 15 dpi (e).

Three construct‐derived transgenic *N. benthamiana* lines (2D, 17 events; A7, five events; and A9, five events) were obtained by *Agrobacterium*‐mediated transformation. To evaluate the antiviral properties of the transgenic *N. benthamiana* plants, the BSCTV agroinfectious clone was injected into 30‐day‐old transgenic T_1_ generation *N. benthamiana* leaves with three replicates for each construct. qPCR analysis was used to quantify BSCTV accumulation in the top leaves of the *N. benthamiana* plants. Compared with the virus accumulation the control, it was reduced by 98%, 99%, and 95% by events 2D‐8, 2D‐9, and 2D‐14, respectively, at 7 days post‐infection (dpi) (Figure 1c), by 70%, 90%, and 90% for events A7‐3, A7‐5, and A7‐9, respectively (Figure 1d), and by 90% and 95% for events A9‐6 and A9‐8 (Figure 1e). Plants infected with BSCTV will exhibit a severely curled and stunted phenotype. Therefore, we also monitored the phenotype of BSCTV‐infected transgenic *N. benthamiana* , negative *N. benthamiana* , and wild‐type *N. benthamiana* during 15 dpi. Transgenic *N. benthamiana* plants from all three constructs grew normally (Figure 2a–c), while negative *N. benthamiana* and wild‐type *N. benthamiana* showed severe curling and stunting at 7 dpi (Figure 2d and Figure S1) and even more severely at 15 dpi (Figure 2e). These results demonstrated that the CRISPR‐Cas12a–derived transgenic *N. benthamiana* inhibited the accumulation of BSCTV and that Cas12a can process fused long mRNA‐pre‐crispr (cr)RNA transcripts into functional mRNA and crRNAs.

### 2.3 CRISPR‐Cas12a Multiplex Gene Editing System Induces Diverse Mutations in BSCTV Genomes

**FIGURE 3:**
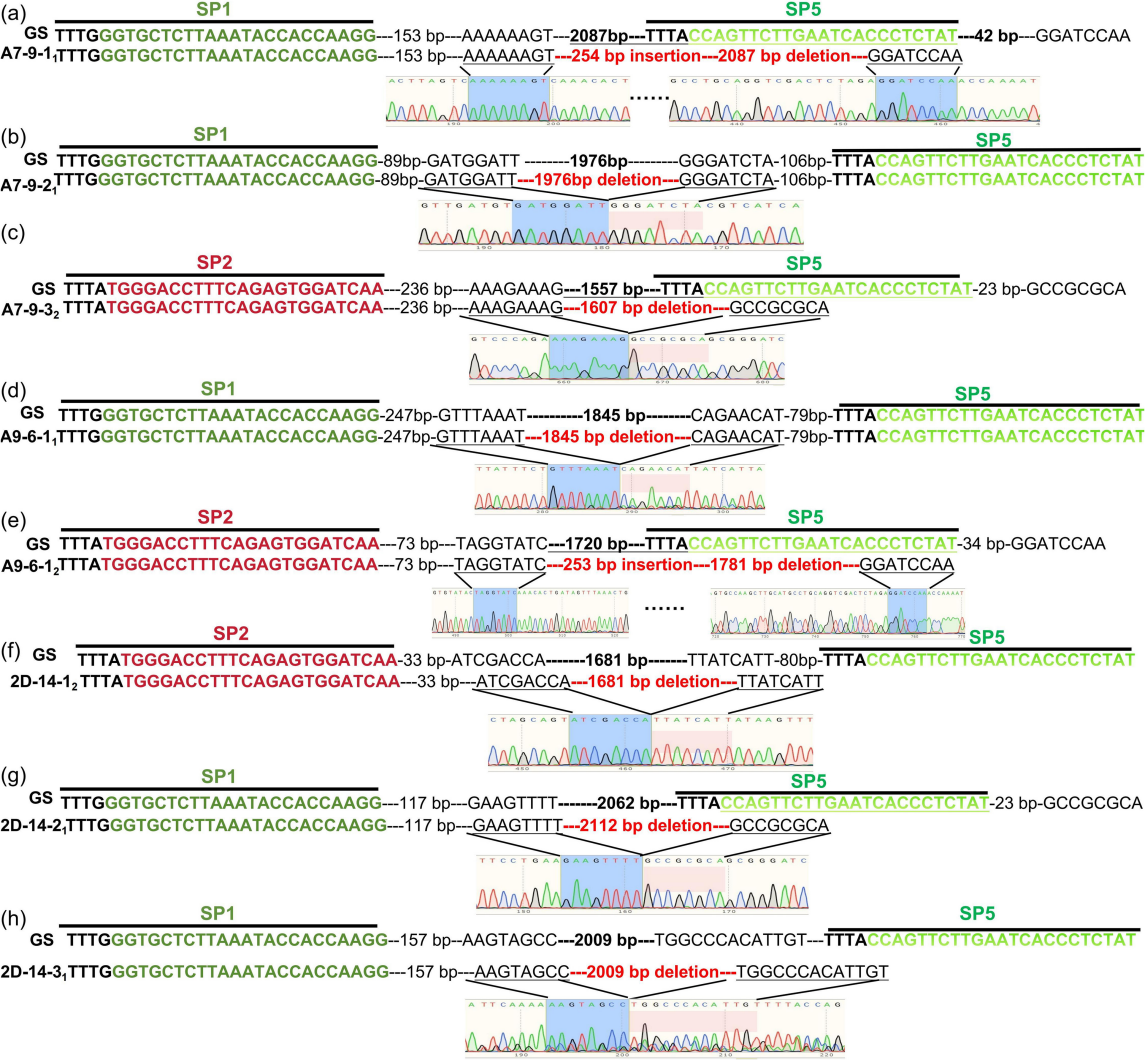
Mutation detection in target sites by sequencing the PCR products. Alignment of DNA sequencing results of the PCR products from transformation event A7‐9 (a–c), A9‐6 (d,e), and 2D‐14 (f–h). gRNA target sites and PAM sequences are overlined. The wild‐type BSCTV genome sequence (GS) is shown at the top, while the sequencing results of the PCR products are displayed in the middle. The original DNA sequencing chromatogram is shown at the bottom. A‐B‐Cn, where A (construct number), B (transformation event number), C (transgenic *Nicotiana benthamiana* plant individual number), and n (PCR product band number). For example, A7‐9‐3_2_ represents the sequencing result of the second band of the PCR product obtained from the third individual of transformation event 7 in the transgenic *N. benthamiana* derived from construct A7.

To determine whether the virus genome in disease‐resistant plants has undergone editing and to evaluate the efficiency of multiplex editing and priority of the six target sites, we designed one pair of primers to detect mutations in all the six target sites in BSCTV genome. DNA samples from the top leaves at 15 dpi, amplified by PCR, showed that each of the three individual transgenic plants derived from the three constructs had more than two bands, except for the negative sample that had one big band (Figure 1f). The diverse band profiles indicate polymorphic mutations in the BSCTV genome, and the bands smaller than 1 kb suggest the possibility of large fragment deletions. We sequenced almost all the bands that we could cut the gel under UV light. Sequencing of the PCR products smaller than 1 kb listed in Table S1, and the detailed description displayed in Figure 3. Three individual transgenic plants of A7‐9 had three types of mutations (Figure 3a–c and Figure S2), 254‐bp insertion and 2087‐bp deletion were detected between SP2 and SP5 in all Three individual transgenic plants (Figure 3a and Figure S2a,b), 1976‐ and 1607‐bp deletion was introduced to the A7‐9‐2 (Figure 3b) and A7‐9‐3 (Figure 3c), and the largest band, approximately 2.5 kb, did not have any mutations in all six targets (Figure S3). Three individual transgenic plants of A9‐6 also had three types of mutations (Figure 3d,e and Figure S2c–e), 253‐bp insertion and 1781‐bp deletion between SP2 and SP5 was the common mutation type (Figure 3e and Figure S2c,e), 1854‐bp deletion was introduced to A9‐6‐1 (Figure 3d), 254‐bp insertion and 2087‐bp deletion was introduced to A9‐6‐3 (Figure S2d), and the negative *N. benthamiana* derived from A9‐6 only had one 2.5‐kb band (Figure 1f) and the sequencing result (Figure S4) was the same as 2.5 kb band of A7‐9 (Figure S3). We also sequenced the 2.5‐kb band of BSCTV‐infected wild type *N. benthamiana* , and no mutations were detected (Figure S5). Three individual transgenic plants of 2D‐14 had five types of mutations (Figure 3f–h and Figure S2f–h), deletions of 1681, 2112, and 2009 bp were introduced to 2D‐14‐1, 2D‐14‐2, and 2D‐14‐3, respectively (Figure 3f–h), 254‐bp insertion and 2087‐bp deletion was introduced to 2D‐14‐1 and 2D‐14‐2 (Figure S2f,g), and 227‐bp insertion and 1645‐bp deletion were introduced to 2D‐14‐3 (Figure S2h).

Taken together, these results indicate that, firstly, our CRISPR/Cas12a multiplex gene editing system can directly disrupt the structure of viral genomes through large fragment deletions. Secondly, the innovative strategy of the mRNA–gRNA nucleic acid complex proved to be highly effective in the gene editing system. Thirdly, both LbCas12a and FnCas12a successfully targeted the SP2 and SP5 sites, enabling the introduction of large fragment deletions to the BSCTV genome.

### 2.4 CRISPR‐Cas12a Multiplex Gene Editing System Introduces Resistance to TMV

**FIGURE 4:**
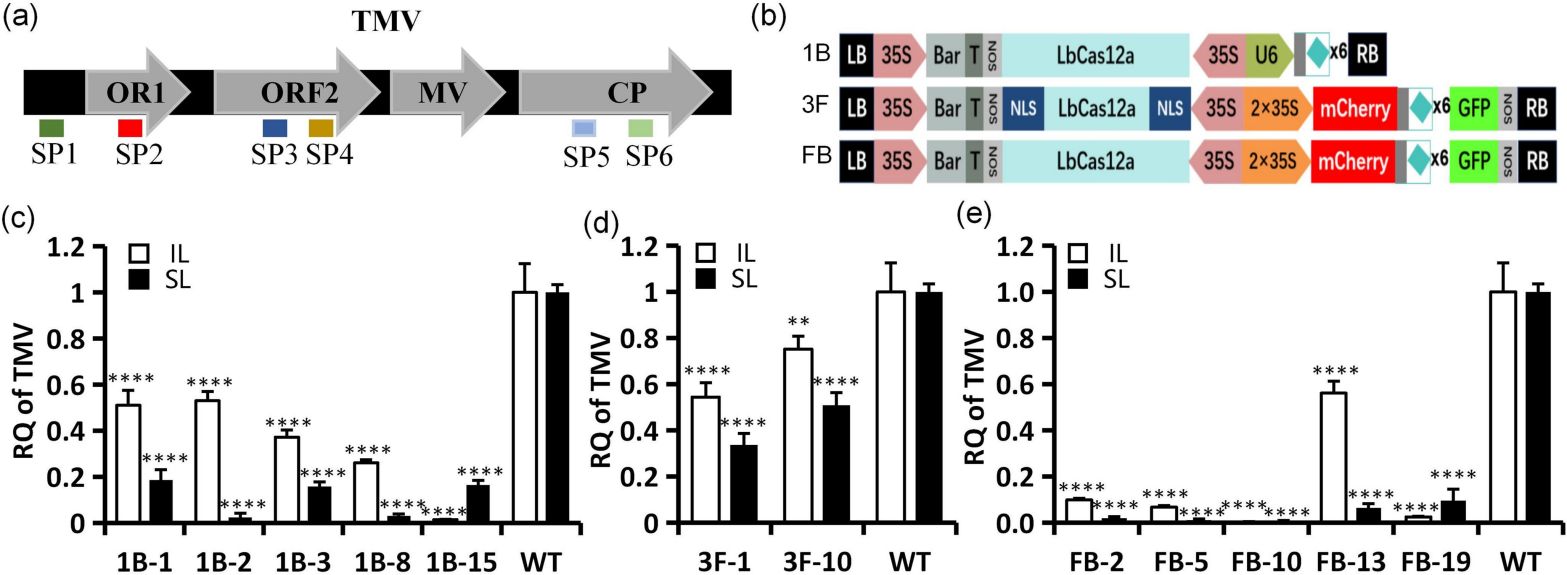
Studies of the strategy of plant anti RNA virus based on CRISPR/Cas12a. (a) Schematic diagram of the TMV genome and gRNA target sites. (b) Schematic diagram of the TMV gene editing vectors. (c–e) Quantification of TMV accumulation by qPCR in transgenic *Nicotiana benthamiana* lines made using constructs 1B (c), FB (d), and 3F (e). RQ of TMV, Relative Quantification of TMV accumulation. IL, inoculated leaves; SL, systemic leaves. Error bars represent the standard deviation; **p < 0.01; ***p < 0.001; ****p < 0.0001. Data are representative of four biological replicates.

TMV is a RNA virus, and its life cycle occurs mainly in the cytoplasm. Theoretically, the CRISPR/Cas12a system also needs to be located in the cytoplasm. However, studies have shown that CRISPR/Cas can confer RNA virus resistance in plants regardless of whether Cas13a is located in the cytoplasm or nucleus using a NLS (Mahas et al. 2019; Zhang et al. 2019). gRNA is a small RNA that can enter and exit the cell nucleus in a specific manner; therefore, it is typically controlled by the U6 promoter. To enhance the efficiency of small RNA entry and exit from the cell nucleus, we have implemented a new approach by fusing a pre‐gRNA to the reporter genes mCherry and green fluorescent protein (GFP), allowing the gRNA to be transported out of the nucleus by the fused mRNA. Therefore, to establish resistance to the invading TMV RNA virus in *N. benthamiana* , we chose to couple the effector protein LbCas12a with six gRNAs targeting the TMV genome (SP1 and SP2 target *ORF1*, SP3 and SP4 target *ORF2*, SP5 targets *MP*, and SP6 targets *CP*) (Figure 4a) to form three vectors (1B, 3F, and FB) (Figure 4b). These vector‐derived T1 transgenic *N. benthamiana* plants were injected with a TMV‐GFP agroinfectious clone containing a GFP‐encoding sequence.

**FIGURE 5:**
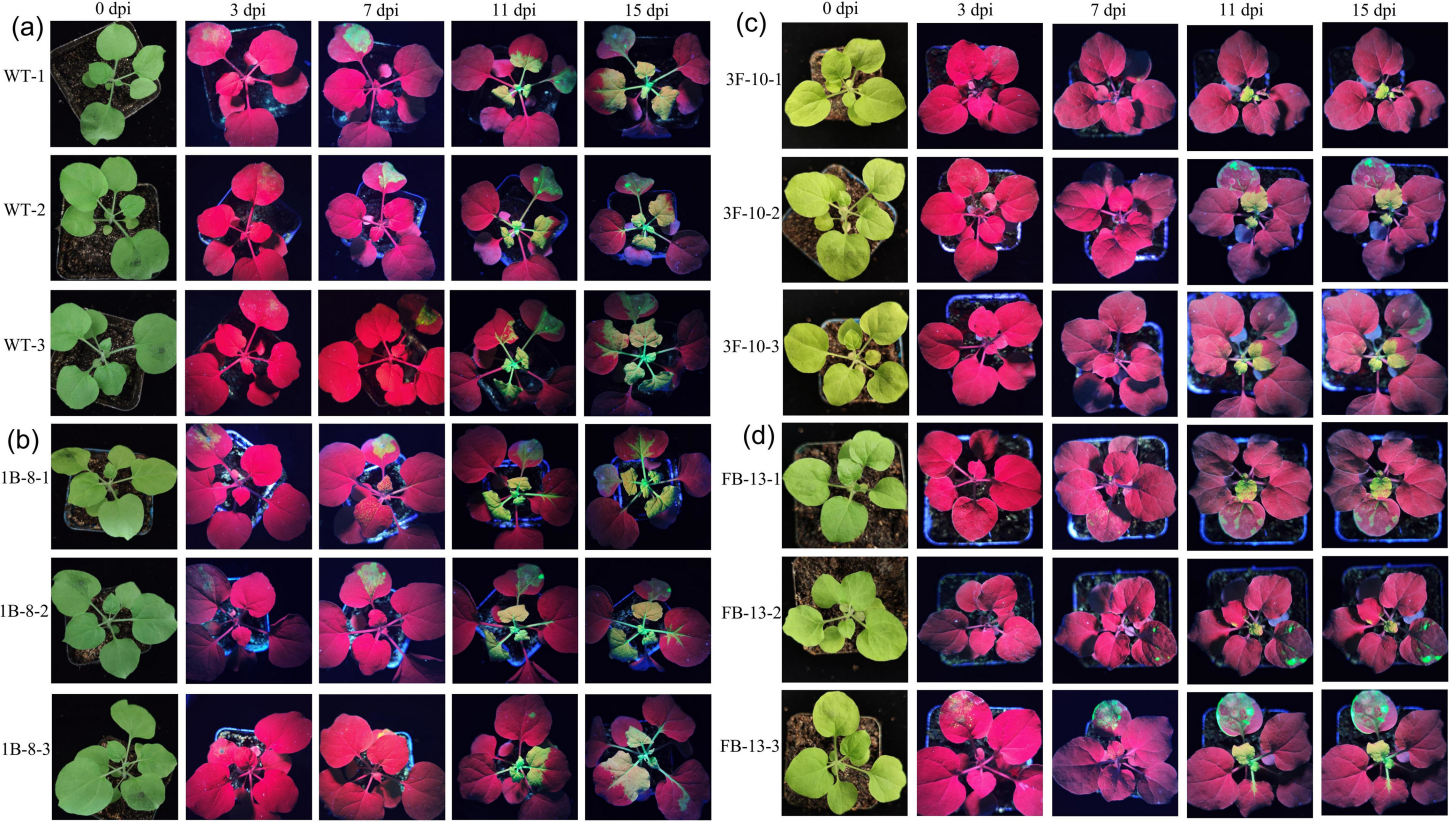
Transgenic *Nicotiana benthamiana* plants established resistance to TMV. The symptoms (GFP signal) of T2 transgenic *N. benthamiana* plants after TMV‐GFP inoculation were monitored. Three T2 individuals from each of the three gene editing constructs derived transgenic *N. benthamiana* and the WT were selected to be imaged under UV light for GFP signal monitoring during 15 dpi. Transgenic *N. benthamiana* from (c,d) mediates efficient interference against TMV than WT (a) and 1B (b) by preventing its systemic spread.

qPCR analysis showed that TMV accumulation was reduced by 50%–70% for four 1B events and by 99% with 1B‐15 in inoculated leaves, and by more than 80% for all five events in 1B in systemic leaves at 7 dpi (Figure 4c). For all five events, the TMV accumulation in both inoculated and systemic leaves was reduced by more than 90%, although just by 40% for FB‐13 in inoculated leaves at 7 dpi (Figure 4e). Transgenic *N. benthamiana* plants derived from constructs 3F displayed weaker antiviral properties than 1B and FB (Figure 4d). The cDNA for two events for each construct‐derived transgenic *N. benthamiana* line was used to detect the mutations in the target sites of the TMV genome; none of them had any mutations (data not shown). We quantified virus accumulation in the transgenic *N. benthamiana* lines by monitoring dynamic changes in GFP fluorescence during 15 dpi (Figure 5). More than three new systemic leaves exhibited strong GFP fluorescence at 11 dpi and expanded to five new systemic leaves at 15 dpi in TMV‐GFP infected WT and 1B transgenic *N. benthamiana* (Figure 5a,b), while only two new systemic leaves exhibited strong GFP fluorescence at 15 dpi in TMV‐GFP infected 3F and FB transgenic *N. benthamiana* (Figure 5c,d). These results indicate that (i) the CRISPR‐Cas12a system can confer resistance to the TMV RNA virus in *N. benthamiana* ; (ii) the strategy of fusing pre‐gRNAs to mRNA more effectively induces TMV resistance.

Taken together, in this study, the CRISPR/Cas12a system was exploited to combat the ssDNA virus BSCTV and ssRNA virus TMV in *N. benthamiana* with high antiviral efficiency in different ways. We explored whether fusing pre‐gRNA to mRNA promoted the efficiency of pre‐gRNA outside the nucleus. Moreover, we have expanded the application of the self‐splicing ability of pre‐crRNA of Cas12a. Finally, we confirmed that the CRISPR‐Cas‐gRNA complex could confer resistance to RNA viruses by hindering their replication. These findings can be used to develop effective antiviral strategies to generate virus‐resistant plants.

## 3 Experimental Procedures

### 3.1 Generation of Transgenic *N. benthamiana*

The *N. benthamiana* plants were cultivated in a greenhouse at 28°C under a 16‐h light/8‐h dark cycle. *Agrobacterium tumefaciens* –mediated transformation was employed to generate transgenic *N. benthamiana* . For the inoculation experiment, T1 transgenic *N. benthamiana* plants and wild‐type *N. benthamiana* with 30‐day‐old leaves need to be selected. Before the inoculation treatment, leaf samples of the transgenic T1 generation *N. benthamiana* plants were taken to extract DNA for verifying the positive and negative traits of the *N. benthamiana* .

### 3.2 Preparation of *Agrobacterium* and Infection of *N. benthamiana* Leaves

The *Agrobacterium* containing BSCTV or TMV‐GFP respectively originated from the research group of Xie Qi at the Chinese Academy of Sciences and the research group of Li Xiangdong at Shandong Agricultural University. The transformed *Agrobacterium* was activated in LB medium containing 50 mg/mL kanamycin and 50 mg/mL rifampicin. The activated bacterial solution was centrifuged at 4°C, 5000 rpm for 10 min. The precipitate was resuspended in MMA (10‐mM 2‐(*N*‐morpholino)ethanesulfonic acid [MES], 10‐mM MgCl_2_, and 0.1‐mM acetosyringone), and the OD600 was adjusted to 0.5. The prepared infection solution was left to stand at room temperature in the dark for 2–4 h. Using a needleless syringe, the infection solution was infiltrated onto the underside of the fourth leaf of *N. benthamiana* . After infection, continuous photography was taken to record the infected and systemic leaves. *N. benthamiana* infected with TMV needed to be photographed under a 365‐nm wavelength light to show GFP fluorescence.

### 3.3 RNA Extraction and qPCR Analysis

In order to determine the accumulation of virus in transgenic *N. benthamiana* , total RNA of 7 dpi systemic leaves was isolated from TMV‐infected transgenic *N. benthamiana* plants. The total RNA was reverse transcribed into cDNA and used as a template for PCR. Quantitative PCR (qPCR) analysis of virus gene expression was performed using SYBR‐Green fluorescent dye. PP2A was used as an internal reference gene with WT as a control, and the relative expression level of the target gene was calculated by comparing the Ct (cycle threshold) values.

### 3.4 Mutation Detection by Genome Sequencing

To test for targeted mutation in the BSCVT genome by the CRISPR‐Cas12a multiplex gene editing system, genomic DNA of transgenic *N. benthamiana* was extracted at 15 dpi. Genomic DNA of *N. benthamiana* was isolated with cetyl trimethyl ammonium bromide (CTAB) buffer. Genomic sequences were amplified using specific primers on the BSCTV genome. The individual fragments isolated from the PCR products upon electrophoresis on a 15% agarose gel were recovered and sequenced.

## Author Contributions

Xiaoqing Liu designed the experiments. Lili Lou and Liqing Miao performed experiments. Lili Lou, Liqing Miao, Xuhui Ma, Jinjin Hu, Suzhen Li, Wenzhu Yang, Shuai Ma, and Rumei Chen analyzed and interpreted the data. Lili Lou, Liqing Miao, and Xiaoqing Liu wrote the manuscript with input from all authors.

## Conflicts of Interest

The authors declare no conflicts of interest.

## Supporting information


**Figure S1** Negative transgenic *N. benthamiana* with BSCTV inoculation
**Figure S2.** Partial sequencing results of the PCR products from transgenic *N. benthamiana* after BSCTV inoculation at 15 dpi
**Figure S3.** Sequencing results of the 2.5 kb PCR products from A7‐9 positive transgenic *N. benthamiana*
**Figure S4.** Sequencing results of the 2.5 kb PCR products from A9‐6 negative transgenic *N. benthamiana*
**Figure S5.** Sequencing results of the 2.5 kb PCR products from wild‐type transgenic *N. benthamiana*
**Table S1.** List of sequenced PCR products

## Data Availability

All data are contained in this manuscript.
