# Supplementary material for: Establishing an Immune System Conferring DNA and RNA Virus Resistance in Plants Using CRISPR/Cas12a Multiplex Gene Editing
Source: Plant Direct. 2025 Apr 7;9(4):e70070. doi: 10.1002/pld3.70070 (PMC11975405; doi:10.1002/pld3.70070)
Supplement: Supplementary file 1 — Figure S1 Negative transgenic N. benthamiana with BSCTV inoculation Figure S2. Partial sequencing results of the PCR products from transgenic N. benthamiana after BSCTV inoculation at 15 dpi Figure S3. Sequencing results of the 2.5 kb PCR products from A7‐9 positive transgenic N. benthamiana Figure S4. Sequencing results of the 2.5 kb PCR products from A9‐6 negative transgenic N. benthamiana Figure S5. Sequencing results of the 2.5 kb PCR products from wild‐type transgenic N. benthamiana Table S1. List of sequenced PCR products [file PLD3-9-e70070-s001.docx]

Establishing an immune system conferring DNA and RNA virus resistance in plants using CRISPR/Cas12a multiplex gene editing

Lili Luo^1^†, Liqing Miao^1^†, Xuhui Ma^1^, Jinjin Hu^2^, Suzhen Li^1^,Wenzhu Yang^1^, Shuai Ma^1^, Rumei Chen^1^ and Xiaoqing Liu^1^*

Supplementary Figures S1-S5+Table S1

**
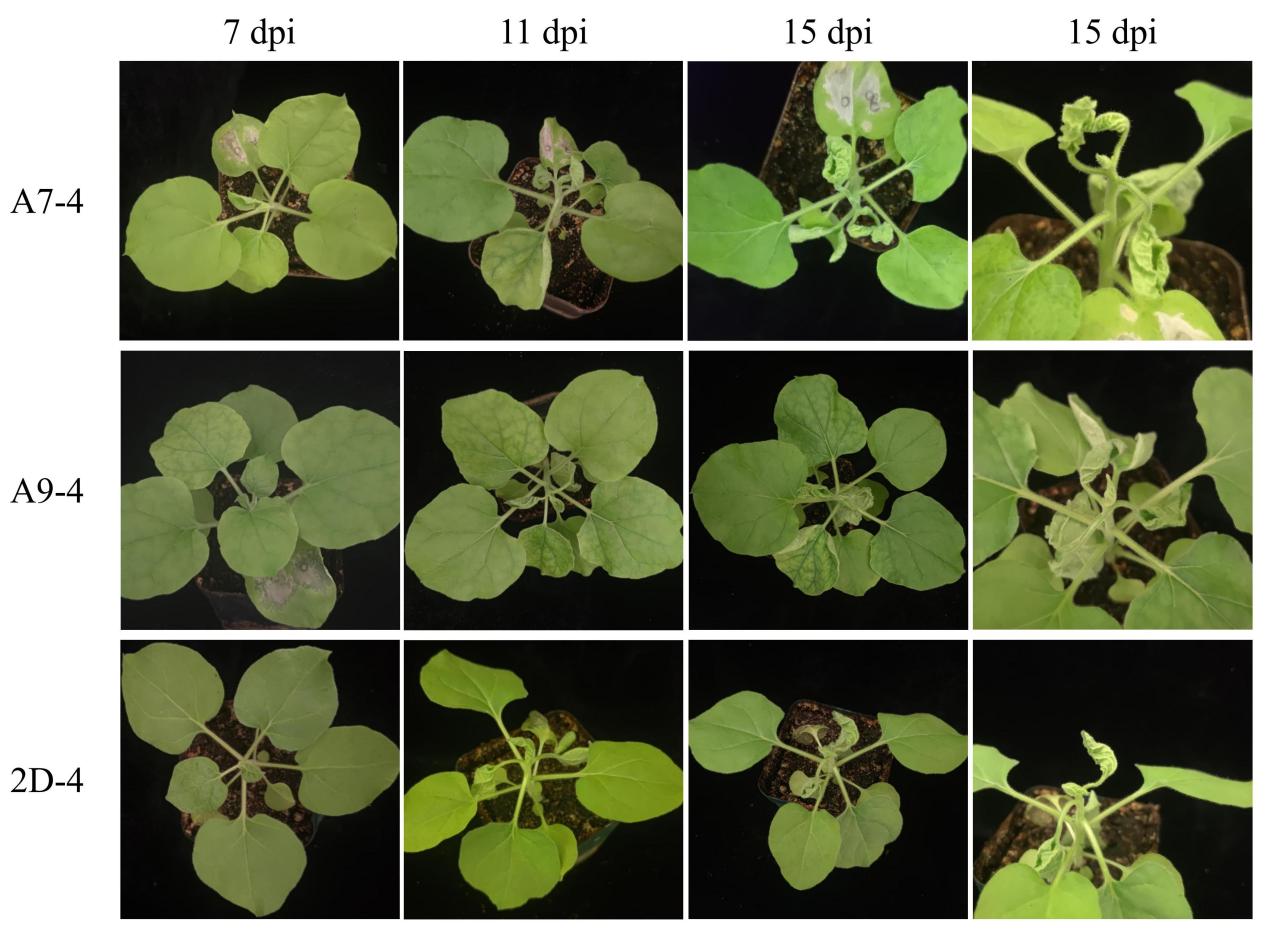
**

**Figure S1. Negative transgenic *N. benthamiana* with BSCTV inoculation**

**
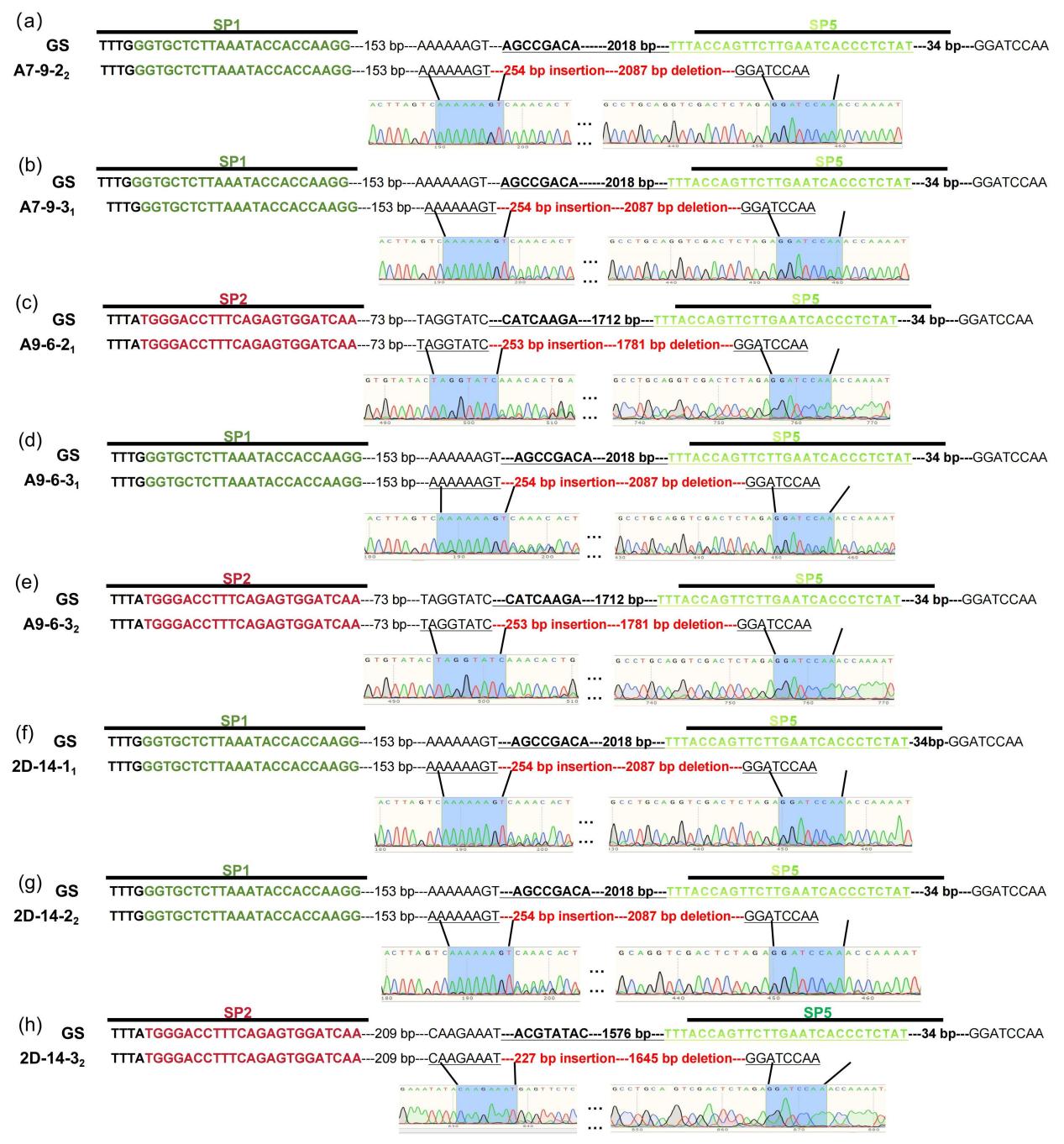
**

**Figure S2. Partial sequencing results of the PCR products from transgenic *N. benthamiana* after BSCTV inoculation at 15 dpi**

**
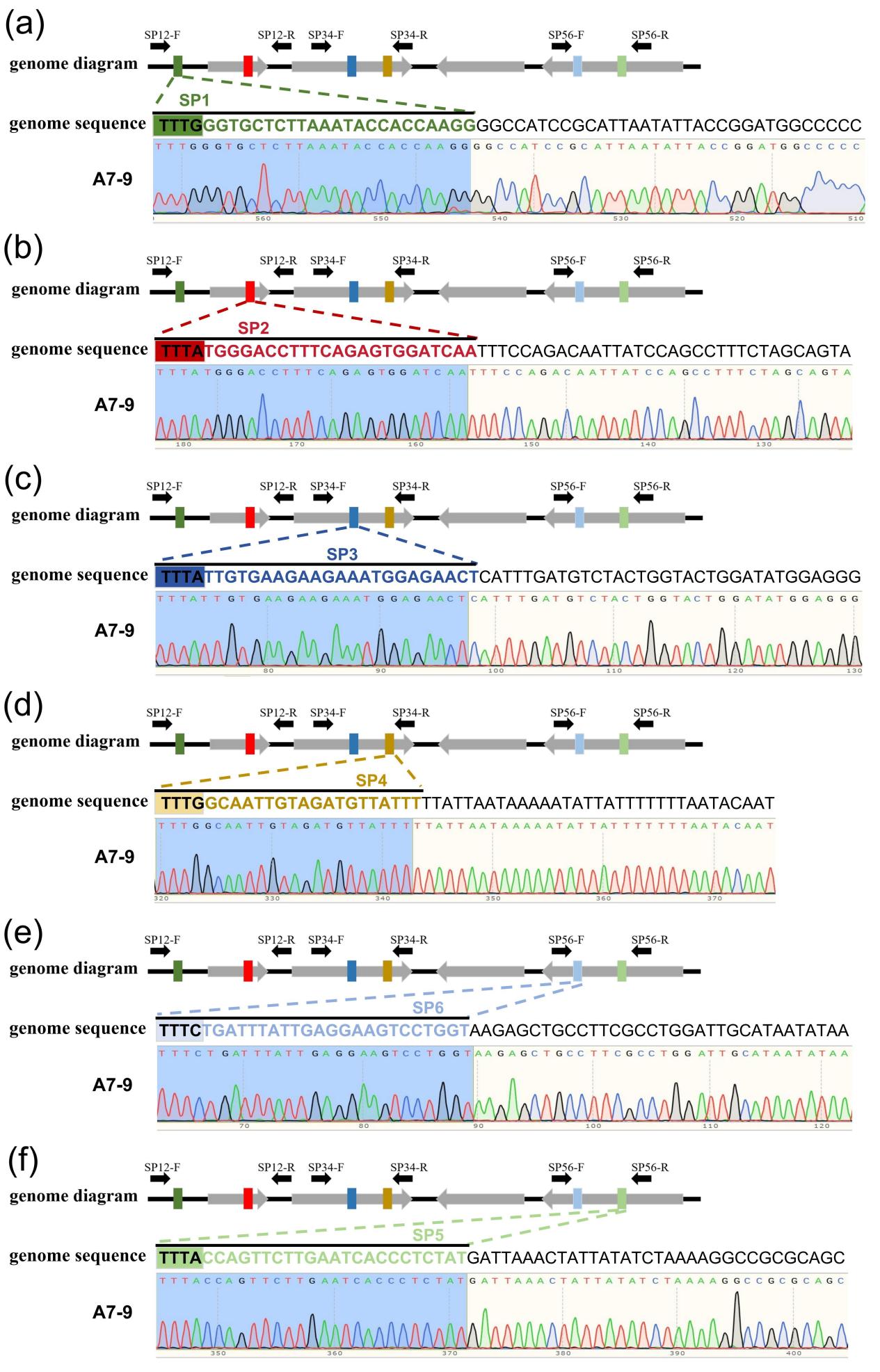
**

**Figure S3. Sequencing results of the 2.5 kb PCR products from A7-9 positive transgenic *N. benthamiana***

**
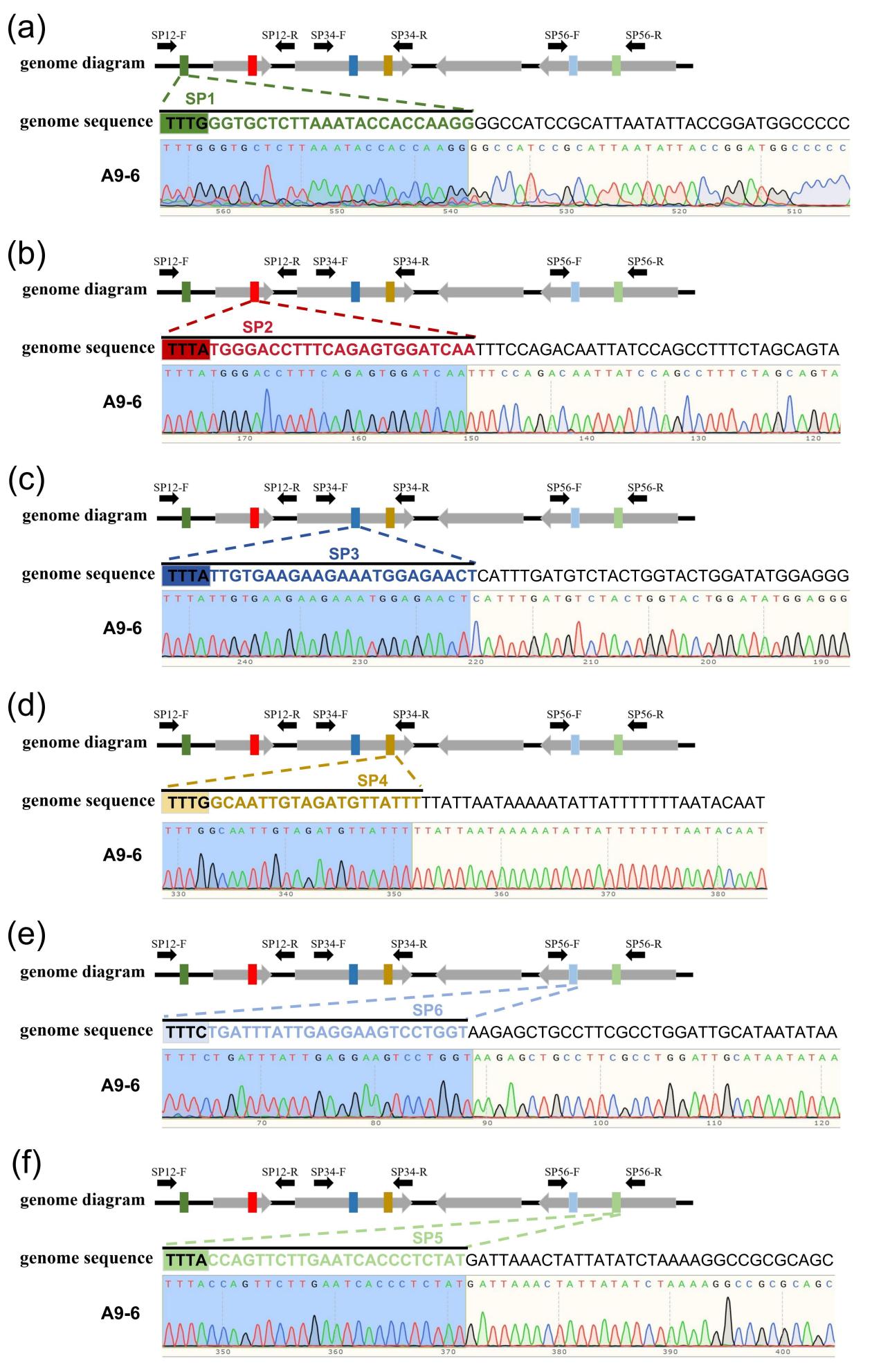
**

**Figure S4. Sequencing results of the 2.5 kb PCR products from A9-6 negative transgenic *N. benthamiana***

**
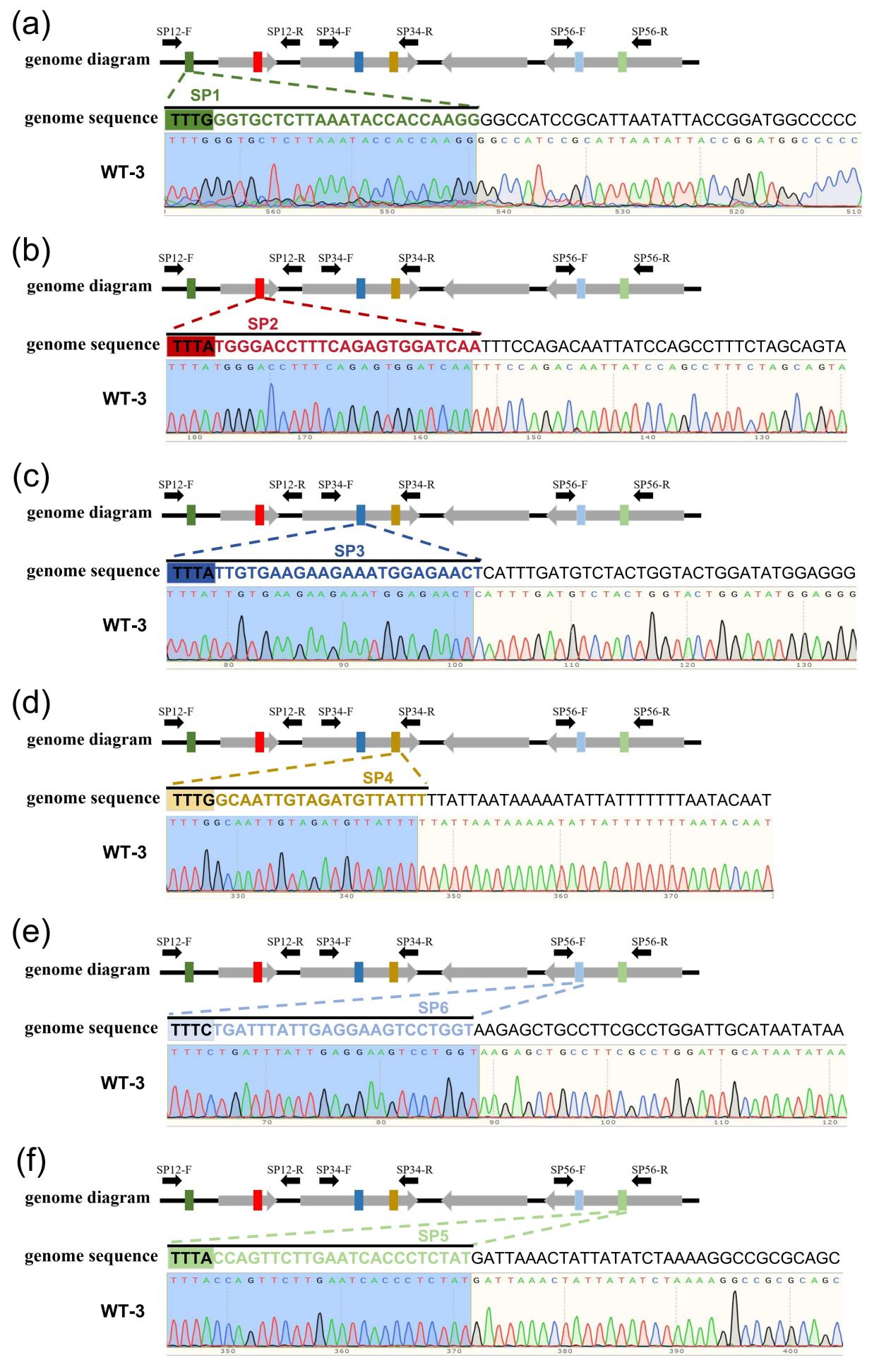
**

**Figure S5. Sequencing results of the 2.5 kb PCR products from wild-type transgenic *N. benthamiana***

**Table S1. List of sequenced PCR products**

| **Event NO.** | **band NO.** | aPCR products |
| --- | --- | --- |
| **A7-9-1** | **1** | 500 bp |
| **A7-9-2** | **1** | 400 bp |
|  | **2** | 500 bp |
| **A7-9-3** | **1** | 500 bp |
|  | **2** | 800 bp |
| **A9-6-1** | **1** | 500 bp |
|  | **2** | 800 bp |
| **A9-6-2** | **1** | 800 bp |
| **A9-6-3** | **1** | 500 bp |
|  | **2** | 800 bp |
| **2D-14-1** | **1** | 500 bp |
|  | **2** | 700 bp |
| **2D-14-2** | **1** | 250 bp |
|  | **2** | 500 bp |
| **2D-14-3** | **1** | 500 bp |
|  | **2** | 800 bp |

^a^The red numbers highlighted in bold indicate the results shown in Figure 3.
